# Frequent utilization of the emergency department for acute exacerbation of chronic obstructive pulmonary disease

**DOI:** 10.1186/1465-9921-15-40

**Published:** 2014-04-10

**Authors:** Kohei Hasegawa, Yusuke Tsugawa, Chu-Lin Tsai, David FM Brown, Carlos A Camargo

**Affiliations:** 1 Department of Emergency Medicine, Massachusetts General Hospital, 326 Cambridge Street, Suite 410, Boston, MA 02114, USA; 2 Harvard Medical School, Boston, MA, USA; 3 Harvard Interfaculty Initiative in Health Policy, Cambridge, MA, USA

**Keywords:** Chronic obstructive pulmonary disease, Exacerbation, Recurrence, Epidemiology, Emergency service, Hospitalization, Mechanical ventilation, Cost, Healthcare utilization

## Abstract

**Background:**

Little is known about patients who frequently visit the emergency department (ED) for acute exacerbation of chronic obstructive pulmonary disease (AECOPD). We aimed to quantify the proportion and characteristics of patients with frequent ED visits for AECOPD and associated healthcare utilization.

**Methods:**

We conducted a retrospective cohort study of adults aged ≥40 years with at least one ED visit for AECOPD between 2010 and 2011, derived from population-based all-payer data of State ED and Inpatient Databases for two large and diverse states: California and Florida. Outcome measures were frequency of ED visits for AECOPD, 30-day ED revisits, subsequent hospitalizations, near-fatal events (AECOPD involving mechanical ventilation), and charges for both ED and inpatient services (available only for Florida) during the year after the first ED visit.

**Results:**

The analytic cohort comprised 98,280 unique patients with 154,736 ED visits for AECOPD. During the 1-year period, 29.4% (95% CI, 29.1%-29.7%) of the patients had two or more (frequent) visits, accounting for 55.2% (95% CI, 54.9%-55.4%) of all ED visits for AECOPD. In the multivariable model, significant predictors of frequent ED visits were age 55–74 years (vs. 40–54 years), male sex, non-Hispanic white or black race, Medicaid insurance (vs. private), and lower median household income (all P < 0.001). At the visit-level, 12.3% of ED visits for AECOPD were 30-day revisit events (95% CI, 12.1%-12.4%). Additionally, 62.8% of ED visits for AECOPD (95% CI, 62.6%-63.0%) resulted in a hospitalization; patients with frequent ED visits comprised 55.5% (95% CI, 55.2%-55.8%) of all hospitalizations. Furthermore, 7.3% (95% CI, 7.3%-7.5%) of ED visits for AECOPD led to a near-fatal event; patients with frequent ED visits accounted for 64.4% (95% CI, 63.5%-65.3%) of all near-fatal events. Total charges for AECOPD were $1.94 billion (95% CI, $1.90-1.97 billion) in Florida; patients with frequent ED visits accounted for $1.07 billion (95% CI, $1.04-1.09 billion).

**Conclusions:**

In this large cohort study, we found that 29% had frequent ED visits for AECOPD and that lower socioeconomic status was significantly associated with a higher frequency of ED visits. Individuals with frequent ED visits for AECOPD accounted for a substantial amount of healthcare utilization and financial burden.

## Background

Chronic obstructive pulmonary disease (COPD) is a serious public health problem in the US. Acute exacerbations of COPD (AECOPD) were responsible for approximately 1.5 million emergency department (ED) visits and 700,000 hospitalizations in 2010 [1]. Furthermore, acute exacerbations accelerate decline in lung function, reduce patient’s quality of life, and increase health care use [2-4]. Indeed, exacerbation management was estimated to account for up to 70% of total direct costs of COPD management [5,6].

In this context, the US government recently identified reducing ED visits for AECOPD as a national objective in *Healthy People 2020*[7]. To develop and implement preventive strategies effectively, identifying patients at risk for future exacerbations is critical. Prior studies have identified risk factors associated with frequent exacerbations (2 or more exacerbations in a year), such as a history of previous exacerbations, disease severity, poor disease-specific health status, gastroesophageal reflex, and non-private insurance status [8-11]. However, these studies were conducted within limited populations (e.g., patients in urban academic centers, and population in one state), thereby limiting generalizability of their results. Despite a substantial burden of COPD-related ED visits in an already stressed healthcare system, there have been no large cohort studies to characterize this high-risk patient population in the US.

To address these knowledge gaps, using large all-payer databases from two geographically dispersed states, we sought to quantify the proportion and characteristics of patients with frequent ED visits for AECOPD and associated healthcare burden, including rates of 30-day ED revisit, hospitalizations, near-fatal events, and hospital charges. A better understanding of these important issues in ED patients with AECOPD may inform potential strategies to improve their COPD management and reduce healthcare spending.

## Methods

### Study design and setting

We conducted a retrospective cohort study using data from the Healthcare Cost and Utilization Project (HCUP) State Emergency Department Databases (SEDD) and State Inpatient Databases (SID). The SEDD includes all treat-and-release and transfer ED visits from short-term, acute-care, nonfederal, community hospitals in participating states. The SID includes all inpatient discharges from short-term, acute-care, nonfederal, general, and other specialty hospitals in participating states, including those admitted from the ED. Taken together, we identified all ED visits regardless of disposition and contained information on short-term outcomes for patients admitted through the ED. Additional details of the SEDD and SID can be found elsewhere [12,13].

In this study, we used the data from California and Florida SEDD and SID in 2010 and 2011. These two states were selected for their geographic distribution, high data quality, and mainly because their databases included unique encrypted patient-level identifiers that enable follow-up of specific patients across years. The institutional review board of our hospital waived review of this study.

### Study population

We identified all adults aged 40 years or older with at least one ED visit for AECOPD in 2010, by using *International Classification of Diseases, Ninth Revision, Clinical Modification (ICD-9-CM)* code for: 1) chronic bronchitis (491.xx), emphysema (492.xx), or chronic airway obstruction (496.xx) in the primary diagnosis field; or 2) acute respiratory failure (518.81, 518.82, or 518.84) listed in the primary diagnosis field and COPD listed as the secondary diagnosis (491.xx, 492.xx, or 496.xx). To minimize the potential misclassification of acute bronchitis as COPD, we did not include bronchitis not specified (490.xx). Likewise, we excluded patients younger than 40 years because they are less likely to have COPD [5]. We also excluded out-of-state residents, and patients who died at the first ED visit or hospitalization during the study period.

### Covariates

The databases contain information on patient characteristics, including demographics (age, sex, and race/ethnicity), primary insurance type, household income, urban–rural status, *ICD-9-CM* diagnosis, and patient comorbidities. The SEDD database also includes ED disposition. The patient characteristics at the first visit were used for the primary analysis. Primary insurance types were categorized into Medicaid, Medicare, private sources, self-pay, and other. Quartile classifications of estimated median household income of residents in the patient’s ZIP Code were examined. These values were derived from the annual ZIP Code-demographic data and included in the SEDD and SID databases. Because these estimate are updated annually, the values ranges for the categories vary by year [12,13]. Urban–rural status of the patient residence was defined according to the National Center for Health Statistics [14]. To adjust for potential confounding by patient-mix, 29 Elixhauser comorbidity measures were derived based on the *ICD-9-CM* codes using the Agency for Healthcare Research and Quality (AHRQ) Comorbidity Software [15]. This risk adjustment tool has been derived and validated extensively [16].

### Outcome measures

The primary outcome measure was the frequency of ED visits for AECOPD in a given year for each patient. The patient’s first ED visit in 2010 was identified as the index ED visit. Each patient was then followed for 365 days after the index visit; then, the total number of ED visits for each patient was summated during the follow-up period, including the index visit.

Other outcome measures of interest were 30-day ED revisits, hospitalizations, near-fatal events, and charges for both ED and inpatient services. Thirty-day ED revisit was defined as an ED visit for AECOPD within 30 days of the previous ED or hospital discharge. Hospitalization was defined as a hospital admission for AECOPD from ED during the year after the index visit. Near-fatal event was defined as an ED visit or hospitalization for AECOPD involving noninvasive or invasive mechanical ventilation [17]; the use of mechanical ventilation was identified using the HCUP *Clinical Classifications Software* code 216. Charges reflect the total facility fees aggregated for a given individual; they are not available in the California datasets. All charges were converted to 2011 US dollars using the medical component of the Consumer Price Index [18].

### Statistical analysis

For the purpose of this analysis, we categorized patients into three ED utilization groups according to the distribution of COPD-related ED visits and previous literature [8]: one ED visit (i.e., index visit only), two ED visits, and three or more ED visits within one year. First, we tested for unadjusted associations between patient-level variables and the frequency of ED visits for AECOPD using chi-square test or Kruskal-Wallis test, as appropriate. The patient-level covariates at the first visit were used for the analysis. Then, we fit multinomial logistic regression models to examine associations between patient-level variables and frequency of ED visits, with one ED visit group as the reference, adjusting for patient-mix using Elixhauser comorbidity measures.

Additionally, we calculated the rate of 30-day ED revisits, hospitalizations, and near-fatal events according to the ED visit utilization. The rates were defined as the total number of respective outcomes within a year of the index ED visit divided by the total number of ED visits for AECOPD. Then, we examined associations between the frequency of ED visits and these rates. We also performed linear regression at the patient-level to examine a linear association between the frequency of ED visits and charges.

In sensitivity analyses, to assess the consistency of associations between the frequency of ED visits and each outcome, we stratified the analysis by state, and included data from Nebraska. Data from Nebraska was not used for the primary analysis as they did not include race/ethnicity. Additionally, to further investigate predictors of a higher frequency of ED visits, a negative binomial regression model with quasi-likelihood estimation was used [19]. This model has advantages that there is no need to define an arbitrary cutoff point and that this model appropriately accounts for statistical over-dispersion [20,21]. All analyses were performed with SAS version 9.3 (SAS Institute, Cary, NC) and results were presented with 95% confidence interval (CI), when appropriate. A two-sided P value <0.05 was considered statistically significant.

## Results

All ED visits for AECOPD made by patients aged 40 years or older in 2010 and 2011 (n = 294,678) were identified in the California and Florida databases. From this population, we sequentially excluded ED visits occurred >365 days after the index visit (n = 23,485), those made by patients who had no ED visits in 2010 (n = 104,510), and those without a valid encrypted patient identifier (n = 6,583). We also excluded ED visits made by patients with out-of-state residence (n = 4,331) or who died at the index ED visit (n = 1033). After these exclusions, the analytic cohort comprised 98,280 unique patients with 154,736 ED visits for AECOPD.

### ED visits for AECOPD

**Figure 1:**
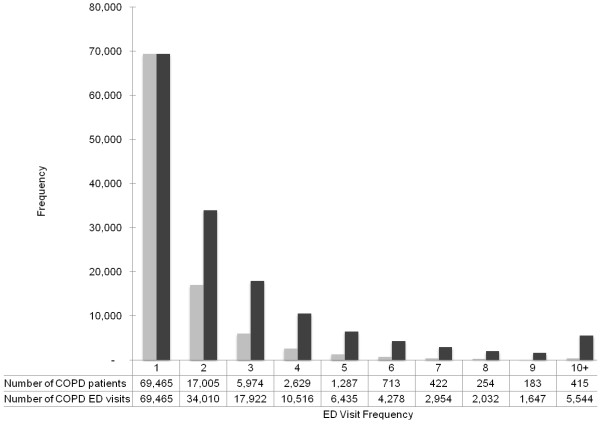
Number of patients and emergency department (ED) visits for acute exacerbation of chronic obstructive pulmonary disease (AECOPD), according to ED visit frequency.

Among this analytic cohort, 69,398 patients (70.6%; 95% CI, 70.3%-70.9%) reported one ED visit during the 1-year study period, while 28,882 patients (29.4%; 95% CI, 29.1%-29.7%) had two or more (frequent) visits. Figure 1 demonstrates the number of patients and ED visits for AECOPD during the study period by ED visit frequency. Patients with frequent visits accounted for 55.2% (95% CI, 54.9%-55.4%) of all ED visits for AECOPD.

**Figure 2:**
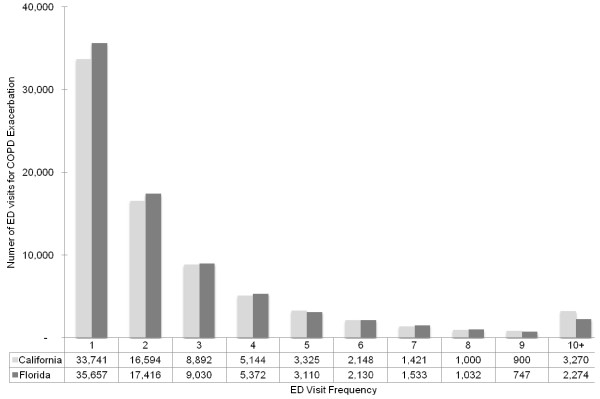
Number of emergency department (ED) visits for acute exacerbation of chronic obstructive pulmonary disease (AECOPD), according to ED visit frequency, stratified by state.

In the sensitivity analysis, the distribution of ED visit frequency was similar across California and Florida (Figure 2), with the comparable findings in Nebraska. For example, 29.7% of patients in California had frequent ED visits and accounted for 55.9% (95% CI, 55.5%-56,2%) of all ED visits; 29.1% patients in Florida had frequent visits and accounted for 54.5% (95% CI, 54.1%-54.8%) of all ED visits; 29.6% patients in Nebraska had frequent ED visits and accounted for 54.0% (95% CI, 52.5%-55.5%) of all ED visits.

### Patient characteristics

Patient demographics, socioeconomic factors, and comorbidities differed across the ED visit frequency groups (Table 1). Compared to patients with one ED visit, patients with a higher frequency of ED visits were more likely to be age 55–74 years, male sex, and non-Hispanic black race. Similarly, several markers of socioeconomic status, such as Medicaid insurance and lower median household income, were associated with a higher frequency of ED visits.

**Table 1:** Demographic and Comorbidities of Patients with Chronic Obstructive Pulmonary Disease (COPD), according to the number of COPD-related emergency department visits in one year

	**1 ED visit**	**2 ED visits**	**≥ 3 ED visits**	
Variables*	**(n = 69,398)**	**(n = 17,005)**	**(n = 11,877)**	**P value**^**†**^
Age (y), median (IQR)	69 (58–79)	69 (59–78)	67 (58–76)	<0.001^‡^
40-54 years	11,922 (17)	2595 (15)	2010 (17)	<0.001
55-64 years	14,807 (21)	3815 (22)	3288 (28)	
65-74 years	17,049 (25)	4575 (27)	3287 (28)	
75-84 years	16,788 (24)	4160 (24)	2441 (21)	
85 years and older	8832 (13)	1860 (11)	851 (7)	
Male sex	30,633 (44)	7632 (45)	5527 (47)	<0.001
Race/ethnicity				<0.001
Non-hispanic white	51,557 (74)	12,746 (75)	8713 (73)	
Non-hispanic black	6349 (9)	1702 (10)	1577 (13)	
Hispanic	7715 (11)	1732 (10)	1068 (9)	
Other	2484 (4)	569 (3)	337 (3)	
Missing	1303 (2)	256 (2)	182 (2)	
Insurance status				
Medicare	46,174 (67)	11,829 (70)	7793 (66)	<0.001
Medicaid	8092 (12)	2417 (14)	2413 (20)	
Private	7931 (11)	1299 (8)	729 (6)	
Self-pay	4322 (6)	71 (4)	431 (4)	
Other	2870 (4)	699 (4)	508 (4)	
Quartiles for median household income of patient’s ZIP code				<0.001
1 (lowest)	22,390 (32)	5749 (34)	4415 (37)	
2	19,307 (28)	4800 (28)	3325 (28)	
3	15,904 (23)	3797 (22)	2497 (21)	
4 (highest)	10,524 (15)	2308 (14)	1372 (12)	
Missing	1273 (2)	351 (2)	268 (2)	
Patient residence				<0.001
Counties of ≥ 1 million population	42,303 (61)	10,245 (60)	7123 (60)	
Counties of 250,000-999,999 population	17,379 (25)	4319 (25)	2983 (25)	
Counties of 50,000-249,999 population	4103 (6)	1053 (6)	750 (6)	
Counties of < 50,000 population	5590 (8)	1335 (8)	956 (8)	
State				0.02
California	33,741 (49)	8297 (49)	5942 (50)	
Florida	35,657 (51)	8708 (51)	5935 (50)	
Any comorbidities	54,633 (79)	13,888 (82)	9644 (81)	<0.001
Selected comorbidities^§^				
Congestive heart failure	13,607 (20)	3831 (23)	2708 (23)	<0.001
Pulmonary circulation disorders	674 (1)	269 (2)	198 (2)	<0.001
Diabetes, uncomplicated	14,813 (21)	3931 (23)	2939 (25)	<0.001
Obesity	7195 (10)	1935 (11)	1437 (12)	<0.001
Depression	6357 (9)	1886 (11)	1479 (12)	<0.001
Psychoses	3233 (5)	1001 (6)	847 (7)	<0.001
Drug abuse	1430 (2)	458 (3)	528 (4)	<0.001

*Abbreviations*: *COPD* chronic obstructive pulmonary disease, *ED* emergency department, *IQR* interquartile range.

*Data were expressed as n (%) unless otherwise indicated.

^†^Unadjusted comparisons were tested using chi-square test unless otherwise indicated.

^‡^Unadjusted comparison was tested using Kruskal-Wallis test.

^§^Comorbidity was defined as at least 1 Elixhauser comorbidity measure.

In a multinomial logistic regression model (Table 2), these patient characteristics remained significant as independent predictors of a higher frequency of ED visits across the frequency groups. For example, non-Hispanic white race (OR, 1.32; 95% CI, 1.23-1.41), non-Hispanic black race (OR, 1.59; 95% CI, 1.45-1.73), Medicaid insurance (OR, 2.92; 95% CI, 2.66-3.20), and lowest quartile for household income (OR, 1.29; 95%CI, 1.21-1.38) were independently associated with three or more ED visits within one year. In addition, certain comorbidities such as congestive heart failure, pulmonary circulation disorders, depression, and psychoses were associated with a higher frequency of ED visits. In the sensitivity analyses, these associations remained statistically significant with stratification by state and the use of negative binomial regression model (Tables 3 and 4).

**Table 2:** Multinomial regression models for factors associated with frequent emergency department visits for acute exacerbation of chronic obstructive pulmonary disease

	**2 (vs. 1) ED visits**		**≥ 3 (vs. 1) ED visits**	
**Variables**	**Odds ratio (95% CI)**	**P value**	**Odds ratio (95% CI)**	**P value**
Age, y		<0.001		<0.001
40-54 years	1 [reference]		1 [reference]	
55-64 years	**1.15 (1.09-1.22)**	<0.001	**1.31 (1.23-1.39)**	<0.001
65-74 years	**1.14 (1.07-1.22)**	<0.001	**1.11 (1.03-1.20)**	0.006
75-84 years	1.05 (0.98-1.12)	0.10	**0.86 (0.79-0.93)**	<0.001
85 years and older	**0.90 (0.83-0.97)**	0.03	**0.58 (0.52-0.64)**	<0.001
Male sex	**1.04 (1.00-1.08)**	0.048	**1.12 (1.07-1.17)**	<0.001
Race/ethnicity				
Non-hispanic white	**1.15 (1.08-1.21)**	<0.001	**1.32 (1.23-1.41)**	<0.001
Non-hispanic black	**1.17 (1.09-1.27)**	<0.001	**1.59 (1.46-1.73)**	<0.001
Hispanic	1 [reference]		1 [reference]	
Other	1.07 (0.96-1.19)	0.29	1.07 (0.96-1.19)	0.33
Insurance status				
Medicare	**1.58 (1.47-1.69)**	<0.001	**2.10 (1.92-2.29)**	<0.001
Medicaid	**1.77 (1.64-1.92)**	<0.001	**2.92 (2.66-3.20)**	<0.001
Private	1 [reference]		1 [reference]	
Self-pay	**1.11 (1.00-1.23)**	0.046	1.05 (0.92-1.20)	0.45
Other	**1.46 (1.32-1.62)**	<0.001	**1.78 (1.57-2.02)**	<0.001
Quartiles for median household income of patient’s ZIP code				
1 (lowest)	**1.13 (1.07-1.20)**	<0.001	**1.29 (1.21-1.38)**	<0.001
2	**1.11 (1.05-1.18)**	<0.001	**1.22 (1.14-1.31)**	<0.001
3	**1.08 (1.01-1.14)**	0.01	**1.14 (1.06-1.22)**	<0.001
4 (highest)	1 [reference]		1 [reference]	
Patient residence				
Counties of ≥ 1 million population	1.07 (1.00-1.15)	0.06	**1.09 (1.01-1.18)**	0.03
Counties of 250,000-999,999 population	1.07 (1.00-1.15)	0.07	1.09 (1.00-1.18)	0.06
Counties of 50,000-249,999 population	1.09 (1.00-1.20)	0.06	1.10 (0.99-1.23)	0.09
Counties of < 50,000 population	1 [reference]		1 [reference]	
State				
California	1.01 (0.98-1.05)	0.44	**1.06 (1.02-1.11)**	0.006
Florida	1 [reference]		1 [reference]	
Selected comorbidities*				
Congestive heart failure	**1.17 (1.11-1.22)**	<0.001	**1.24 (1.17-1.30)**	<0.001
Pulmonary circulation disorders	**1.41 (1.22-1.64)**	<0.001	**1.39 (1.17-1.64)**	<0.001
Diabetes, uncomplicated	**1.05 (1.01-1.10)**	0.02	**1.13 (1.07-1.19)**	<0.001
Obesity	0.99 (0.94-1.05)	0.79	0.97 (0.91-1.03)	0.31
Depression	**1.21 (1.14-1.28)**	<0.001	**1.38 (1.30-1.47)**	<0.001
Psychoses	**1.17 (1.09-1.27)**	<0.001	**1.27 (1.17-1.38)**	<0.001
Drug abuse	**1.22 (1.09-1.37)**	<0.001	**1.66 (1.48-1.85)**	<0.001

*Abbreviations*: *COPD* chronic obstructive pulmonary disease, *ED* emergency department, *CI* confidence interval.

Bold results are statistically significant.

*Non-significant Elixhauser comorbidity measures across the frequency groups included peripheral vascular disease, hypertension, neurological disorders, diabetes with complications, liver disease, peptic ulcer disease, acquired immunodeficiency syndrome, lymphoma, solid tumor without metastasis, coagulopathy, fluid and electrolyte disorders, anemia, and alcohol abuse.

**Table 3:** Multinomial regression models for factors associated with frequent emergency department visits for acute exacerbation of chronic obstructive pulmonary disease, stratified by state

	**California**				**Florida**			
	**2 (vs. 1) ED visits**		**≥ 3 (vs. 1) ED visits**		**2 (vs. 1) ED visits**		**≥ 3 (vs. 1) ED visits**	
**Variables**	**Odds ratio (95% CI)**	**P value**	**Odds ratio (95% CI)**	**P value**	**Odds ratio (95% CI)**	**P value**	**Odds ratio (95% CI)**	**P value**
Age, y								
40-54 years	1 [reference]		1 [reference]		1 [reference]		1 [reference]	
55-64 years	**1.20 (1.11-1.31)**	<0.001	**1.32 (1.21-1.45)**	<0.001	**1.11 (1.03-1.21)**	0.008	**1.28 (1.17-1.41)**	<0.001
65-74 years	**1.17 (1.07-1.29)**	0.001	**1.16 (1.04-1.29)**	0.008	**1.13 (1.03-1.23)**	0.01	1.05 (0.95-1.17)	0.34
75-84 years	**1.15 (1.04-1.27)**	0.007	0.90 (0.80-1.01)	0.08	0.98 (0.89-1.08)	0.73	**1.81 (0.73-0.91)**	<0.001
85 years and older	0.99 (0.88-1.11)	0.83	**0.61 (0.53-0.70)**	<0.001	**0.85 (0.76-0.96)**	0.007	**0.54 (0.47-0.63)**	<0.001
Male sex	**1.05 (1.00-1.10)**	0.048	**1.13 (1.06-1.19)**	<0.001	1.03 (0.98-1.08)	0.30	**1.11 (1.05-1.18)**	<0.001
Race/ethnicity								
Non-hispanic white	**1.15 (1.06-1.25)**	0.001	**1.32 (1.20-1.46)**	<0.001	**1.13 (1.03-1.23)**	0.004	**1.30 (1.17-1.44)**	<0.001
Non-hispanic black	**1.24 (1.11-1.38)**	0.001	**1.71 (1.51-1.93)**	<0.001	**1.12 (1.01-1.25)**	0.04	**1.49 (1.31-1.69)**	<0.001
Hispanic	1 [reference]		1 [reference]		1 [reference]		1 [reference]	
Other	1.23 (1.00-1.28)	0.06	1.16 (0.99-1.35)	0.06	0.85 (0.65-1.10)	0.21	0.80 (0.57-1.12)	0.20
Insurance status								
Medicare	**1.55 (1.41-1.71)**	<0.001	**1.88 (1.67-2.11)**	<0.001	**1.63 (1.47-1.81)**	<0.001	**2.41 (2.11-2.75)**	<0.001
Medicaid	**1.65 (1.48-1.83)**	<0.001	**2.58 (2.29-2.92)**	<0.001	**1.96 (1.75-2.20)**	<0.001	**3.38 (2.93-3.89)**	<0.001
Private	1 [reference]		1 [reference]		1 [reference]		1 [reference]	
Self-pay	1.10 (0.95-1.28)	0.21	1.05 (0.87-1.27)	0.62	1.13 (0.98-1.29)	0.09	1.11 (0.92-1.33)	0.29
Other	**1.34 (1.14-1.57)**	<0.001	**1.34 (1.10-1.62)**	0.004	**1.58 (1.37-1.81)**	<0.001	**2.25 (1.90-2.67)**	<0.001
Quartiles for median household income of patient’s ZIP code								
1 (lowest)	**1.24 (1.13-1.37)**	<0.001	**1.18 (1.09-1.28)**	<0.001	**1.10 (1.01-1.19)**	0.03	**1.22 (1.11-1.34)**	<0.001
2	**1.22 (1.12-1.32)**	<0.001	**1.24 (1.13-1.37)**	<0.001	1.03 (0.95-1.11)	0.49	**1.19 (1.08-1.31)**	<0.001
3	**1.12 (1.03-1.21)**	0.001	**1.20 (1.09-1.33)**	<0.001	1.04 (0.95-1.13)	0.40	1.07 (0.97-1.19)	0.19
4 (highest)	1 [reference]		1 [reference]		1 [reference]		1 [reference]	
Patient residence								
Counties of ≥ 1 million population	1.00 (0.89-1.12)	0.99	0.97 (0.86-1.11)	0.69	**1.10 (1.01-1.20)**	0.03	**1.16 (1.04-1.28)**	0.005
Counties of 250,000-999,999 population	1.06 (0.94-1.20)	0.34	1.02 (0.89-1.17)	0.78	1.07 (0.98-1.17)	0.16	1.09 (0.98-1.22)	0.10
Counties of 50,000-249,999 population	1.01 (0.87-1.06)	0.92	1.04 (0.89-1.22)	0.65	**1.16 (1.02-1.32)**	0.02	1.10 (0.94-1.28)	0.23
Counties of < 50,000 population	1 [reference]		1 [reference]		1 [reference]		1 [reference]	
Selected comorbidities								
Congestive heart failure	**1.16 (1.09-1.23)**	<0.001	**1.27 (1.18-1.36)**	<0.001	**1.18 (1.11-1.26)**	<0.001	**1.22 (1.13-1.31)**	<0.001
Pulmonary circulation disorders	**1.51 (1.22-1.86)**	<0.001	**1.34 (1.04-1.73)**	0.02	**1.29 (1.05-1.60)**	0.02	**1.37 (1.09-1.73)**	0.008
Diabetes, uncomplicated	1.02 (0.96-1.09)	0.47	**1.14 (1.06-1.23)**	<0.001	**1.08 (1.02-1.15)**	0.009	**1.12 (1.05-1.20)**	<0.001
Obesity	1.01 (0.92-1.10)	0.85	0.96 (0.87-1.06)	0.42	0.98 (0.91-1.06)	0.60	0.97 (0.89-1.06)	0.50
Depression	**1.22 (1.11-1.33)**	<0.001	**1.39 (1.26-1.54)**	<0.001	**1.19 (1.10-1.28)**	<0.001	**1.37 (1.26-1.49)**	<0.001
Psychoses	**1.19 (1.07-1.32)**	0.001	**1.26 (1.13-1.42)**	<0.001	**1.16 (1.04-1.29)**	0.01	**1.28 (1.14-1.45)**	<0.001
Drug abuse	**1.27 (1.10-1.47)**	0.001	**1.72 (1.49-1.97)**	<0.001	1.16 (0.97-1.39)	0.10	**1.55 (1.30-1.86)**	<0.001

*Abbreviations*: *COPD* chronic obstructive pulmonary disease, *ED* emergency department, *CI* confidence interval.

Bold results are statistically significant.

**Table 4:** Negative binomial regression model for factors associated with frequency of emergency department visits for acute exacerbation of chronic obstructive pulmonary disease

**Variables**	**Risk ratio (95% CI)**	**P value**
Age, y		
40-54 years	1 [reference]	
55-64 years	**1.07 (1.06-1.09)**	<0.001
65-74 years	**1.02 (1.00-1.03)**	0.04
75-84 years	**0.94 (0.92-0.96)**	<0.001
85 years and older	**0.86 (0.85-0.88)**	<0.001
Male sex		
Race/ethnicity		
Non-hispanic white	**1.08 (1.06-1.09)**	<0.001
Non-hispanic black	**1.15 (1.13-1.17)**	<0.001
Hispanic	1 [reference]	
Other	1.01 (0.98-1.03)	0.62
Insurance status		
Medicare	**1.21 (1.19-1.23)**	<0.001
Medicaid	**1.34 (1.32-1.36)**	<0.001
Private	1 [reference]	
Self-pay	1.01 (0.99-1.03)	0.45
Other	**1.13 (1.10-1.16)**	<0.001
Quartiles for median household income of patient’s ZIP code		
1 (lowest)	**1.06 (1.05-1.08)**	<0.001
2	**1.05 (1.03-1.06)**	<0.001
3	**1.03 (1.01-1.04)**	<0.001
4 (highest)	1 [reference]	
Patient residence		
Counties of ≥ 1 million population	**1.03 (1.01-1.05)**	<0.001
Counties of 250,000-999,999 population	**1.04 (1.02-1.05)**	<0.001
Counties of 50,000-249,999 population	**1.03 (1.01-1.06)**	0.007
Counties of < 50,000 population	1 [reference]	
State		
California	**1.02 (1.02-1.03)**	<0.001
Florida	1 [reference]	
Selected comorbidities		
Congestive heart failure	**1.05 (1.04-1.06)**	<0.001
Pulmonary circulation disorders	**1.13 (1.09-1.17)**	<0.001
Diabetes, uncomplicated	**1.04 (1.03-1.05)**	<0.001
Obesity	**0.97 (0.96-0.99)**	<0.001
Depression	**1.08 (1.07-1.10)**	<0.001
Psychoses	**1.08 (1.06-1.10)**	<0.001
Drug abuse	**1.18 (1.15-1.21)**	<0.001

*Abbreviations*: *COPD* chronic obstructive pulmonary disease, *ED* emergency department *CI* confidence interval.

Bold results are statistically significant.

### COPD 30-day ED revisits, hospitalizations, and near-fatal events

Table 5 summarizes clinical outcomes at the visit-level. Overall, 12.3% (95% CI, 12.1%-12.4%) of ED visits for AECOPD were 30-day revisit events. Patients with a higher frequency of ED visits were more likely to develop a 30-day ED revisit (Table 5), regardless of state (all P < 0.001; Table 6).

**Table 5:** Outcomes of patients with Chronic Obstructive Pulmonary Disease (COPD), according to the number of COPD-related emergency department visits in one year

**Variables**	**1 ED visit**	**2 ED visits**	**≥ 3 ED visits**	**P value**
Number of ED visits for COPD, no.	69,398	34,010	51,328	–
30-day ED revisit rate for COPD, % (95% CI)*	–	10.5 (10.1-10.8)	30.0 (29.6-30.4)	<0.001
Hospitalization rate for COPD, % (95% CI)	62.3 (62.0-62.7)	64.9 (64.4-65.4)	62.1 (61.6-62.5)	<0.001
Near-fatal event rate, % (95% CI)	5.8 (5.6-6.0)	8.2 (7.9-8.5)	8.8 (8.6-9.1)	<0.001

*Abbreviations*: *COPD* chronic obstructive pulmonary disease *ED* emergency department, *IQR* interquartile range, *CI* confidence interval.

*Excluding the index ED visits and hospitalizations.

**Table 6:** Outcomes of chronic obstructive pulmonary disease patients, according to the number of emergency department visits, stratified by state

**Variables**	**1 ED visit**	**2 ED visits**	**≥ 3 ED visits**	**P value**
**California**				
Number of ED visits for COPD, no.	33,741	16,594	26,100	–
30-day ED revisit rate for COPD, % (95% CI)*	–	11.1 (10.6-11.6)	31.7 (31.2-32.3)	<0.001
Hospitalization rate for COPD, % (95% CI)	55.7 (55.2-56.2)	54.6 (53.9-55.4)	54.5 (53.9-55.1)	<0.001
Near-fatal event rate, % (95% CI)	6.8 (6.5-7.1)	9.2 (8.7-9.6)	9.6 (9.2-9.9)	<0.001
**Florida**				
Number of ED visits for COPD, no.	35,657	17,416	25,228	–
30-day ED revisit rate for COPD, % (95% CI)*	–	9.8 (9.4-10.3)	28.2 (27.6-28.8)	<0.001
Hospitalization rate for COPD, % (95% CI)	68.6 (68.1-69.0)	71.7 (71.0-72.3)	69.9 (69.3-70.4)	<0.001
Near-fatal event rate, % (95% CI)	4.9 (4.7-5.1)	7.3 (6.9-7.7)	9.9 (9.5-10.3)	<0.001
**Nebraska**				
Number of ED visits for COPD, no.	2009	1060	1301	
30-day ED revisit rate for COPD, % (95% CI)*	–	8.6 (7.0-10.4)	27.2 (24.8-29.7)	<0.001
Hospitalization rate for COPD, % (95% CI)	52.5 (50.3-54.7)	56.6 (53.6-59.6)	53.0 (50.2-55.7)	0.08
Near-fatal event rate, % (95% CI)	6.9 (5.8-8.1)	7.9 (6.4-9.7)	11.2 (9.6-13.1)	<0.001

*Abbreviations*: *COPD* chronic obstructive pulmonary disease, *ED* emergency department, *IQR* interquartile range *CI* confidence interval.

*Excluding the index ED visits and hospitalizations.

Overall, approximately two-thirds of ED visits (62.8%; 95% CI, 62.6%-63.0%) resulted in a hospitalization. Hospitalization rates were greater than 60% across the ED frequency groups and highest with the patients with two ED visits (Table 5). Patients with frequent ED visits accounted for 55.5% (95% CI, 55.2%-55.8%) of total hospitalizations for AECOPD.

Additionally, 7.3% (95% CI, 7.2%-7.5%) of ED visits led to a near-fatal event. Patients with a higher frequency of ED visits had a higher chance of near-fatal event during the 1-year period, regardless of state (all P < 0.001; Tables 5 and 6). Patients with frequent ED visits accounted for 64.4% (95% CI, 63.5%-65.3%) of near-fatal events.

### Hospital charges for AECOPD in Florida

**Figure 3:**
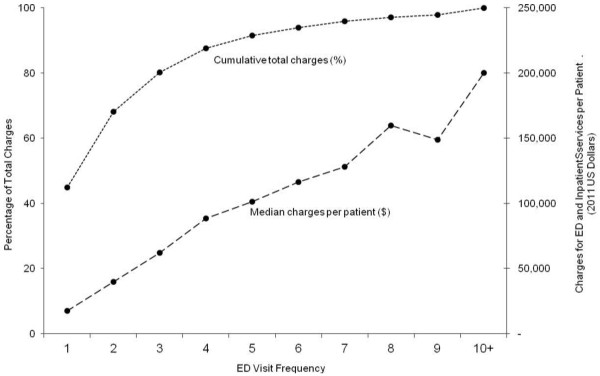
**Median and cumulative charges for emergency department (ED) and inpatient services for acute exacerbation of chronic obstructive pulmonary disease (AECOPD), according to ED visit frequency.** There was a significant linear association between frequency of ED visits and charges per patient (P < 0.001).

The total charges for ED and inpatient services for AECOPD were $1.94 billion (95% CI, $1.90-1.97 billion) in Florida. Patients with frequent ED visits accounted for the majority of total charges ($1.07 billion; 95% CI, $1.04-1.09 billion); this was driven by a significant linear association between number of ED visits and charges per patient (P < 0.001; Figure 3). In addition, recurrent visits after the index ED visits accounted for 37.1% of total charges ($719 million; 95% CI, $709-731 million).

## Discussion

Our study of 98,280 COPD patients showed that 29% of the patients had frequent ED visits for AECOPD (2 or more ED visits in a year). The societal burden of this population with COPD – as measured by ED visits, hospitalizations, and near-fatal events – was large. Indeed, this high-risk population accounted for 55% of all ED visits, 56% of all COPD hospitalizations, 64% of near-fatal events, all contributing to substantial healthcare spending. We also found that male sex, non-Hispanic white and black race, and lower socioeconomic status were significantly associated with a higher frequency of ED visits. These findings inform potential strategies to reduce the large public health burden from AECOPD.

### Proportion of frequent ED visits for AECOPD

To date, most clinical research on patients with AECOPD has focused on predicting and preventing acute relapse and 30-day readmission [22-24]. However, the predictors of acute relapse are not necessarily the predictors of frequent ED visits over longer periods. Conceptually, relapse is more likely to be associated with acute or medical factors, such as severity of exacerbation and quality of care at the index ED visit or hospitalization [24]. In contrast to acute relapse, recurrent ED visits for AECOPD over the course of a longer period of time, such as 1 year in our study, have a broader meaning because it may reflect a failure of less costly and more prevention-oriented COPD care.

A multicenter ED-based study in the early 2000s found that 64% of ED patients with AECOPD self-reported frequent ED visits within past year [11]. By contrast, a claims data study from North Carolina found that 28% of ED patients with AECOPD had frequent ED visits in the late 2000s [10]. Between 2010 and 2011, we demonstrated that 29% of ED patients with AECOPD had frequent ED visits in the two large, geographically-dispersed states, with a comparable percentage in a third state.

The reasons for the differences in the proportion of frequent ED visits for AECOPD among the studies are unclear and likely multifactorial. The population of the earlier multicenter study was limited to urban teaching hospitals that might have disproportionately high COPD morbidity, and thereby led to more frequent ED visits. Alternatively, the observed lower proportion of frequent ED visits might reflect declines in the rate of severe AECOPD over the past decade [1,25]. Secular changes in multiple factors might have contributed to such a potential improvement, including favorable changes in personal health behavior (e.g., smoking), self-management education, access to longitudinal care, hospital-at-home care, and dissemination of evidence-based therapy. For example, the release of Global Initiatives for Chronic Obstructive Lung Disease guidelines in 2001, 2006, and 2011 may have helped reduce the rate of AECOPD [26]. Indeed, after the publication of the first guidelines, researchers reported increases in the use of long-acting beta-agonists with corticosteroids [27] and influenza vaccinations [28] as well as reductions in smoking rate [29]. However, although COPD care has improved, the use of recommended medication combinations and stage-appropriate treatment still has ample room for improvement [30]. The observed lower proportion of frequent ED visits for AECOPD in 2010–2011 supports cautious optimism that COPD morbidity can be prevented and the societal burden reduced. Yet, considering the previous knowledge that only half of patients with AECOPD seek care in the ED or outpatient clinics [31], the large remaining burden underscores the need for continued secondary prevention efforts among patients with COPD.

### Characteristics of patients with frequent ED visits for AECOPD

We were also struck by the disproportionate socioeconomic disparity in frequency of ED visits for AECOPD. In the present study, we found that adults at highest risk of frequent ED visits were more likely to have public insurance, and to have lower socioeconomic status. These results support prior reports of higher rates of frequent ED visits for COPD among these vulnerable populations [10,11]. Although the precise role of lower socioeconomic status to this disparity in COPD healthcare utilizations is unclear [5], the asthma literature would suggest that differences in health beliefs, less self-management education, and limited access to preventive and specialist care in this population might lead to a heavier reliance on episodic symptom treatment and emergency care [32]. Other potential factors that might explain the difference in ED use across socioeconomic status include degree of past and current smoking, living conditions, outpatient treatment, and COPD-specific disease severity [5,33]. The pathway through which socioeconomic status affects healthcare utilization is undoubtedly complex; further investigation is warranted to better understand the underlying reasons for this disparity in ED utilization among patients with AECOPD.

### Burden of patients with frequent ED visits for AECOPD

Consistent with the previous study from North Carolina [10], we observed substantial patient morbidity, as measured by ED visits and hospitalizations for AECOPD. Additionally, our study extended prior research by documenting relatively high use of intensive care resources and high healthcare spending in frequent ED users with AECOPD. Indeed, patients with frequent ED visits accounted for only 29% of all patients; however, they comprised 64% of mechanical ventilation use and 55% of the total charges for ED and inpatient services.

COPD is designated as an ambulatory care sensitive condition by the AHRQ, among other conditions [34]; ED visits for AECOPD can often be prevented by evidence-based outpatient care (e.g., smoking cessation, influenza and pneumococcal vaccines, knowledge of current therapy including inhaler technique, and evidence-based pharmacologic treatment) [5]. Therefore, organized efforts to develop and implement systems of care are imperative to lower COPD adverse outcomes and healthcare expenditures. If recurrent ED visits could be prevented in this study, there would have been approximately 56,456 prevented ED visits and 35,520 prevented hospitalizations in these two states alone. In terms of cost, this would have saved 719 million dollars in Florida alone.

Although these administrative data are unable to explore more granular aspects of COPD care, our findings can better inform a system of care for patients with AECOPD. At the individual patient level, the use of patient characteristics to identify patients at high-risk for frequent AECOPD remains limited [35]. The present findings underscore the importance of translating high-quality research into the risk stratification, coupled with dissemination of these finding to improve care for patients with COPD. At the health system level, our observation should facilitate further work on how to reduce the enormous and uneven public heath burden by targeting the population at greatest risk for integrated research, health policy, and community action.

### Potential limitations

Our study should be viewed in the context of several potential limitations. Our data were not derived from a sample of the national COPD population. However, the data from two geographically diverse states included all ED visits and hospitalizations for approximately 19% of the US population [36]; the age and sex distributions of the ED visits mirrored a national surveillance of COPD-related ED visits [1]. Moreover, our findings persisted across the two geographically dispersed states, with the comparable findings in a third state, suggesting a potential generalizability at the nation-level. Secondly, as with any studies using administrative data, there may be some misclassification of medical claims. However, HCUP data are highly accurate, rigorously tested, and widely used to estimate diagnoses and visit frequency [37,38]. Thirdly, our data do not include information on outpatient treatment and COPD-specific disease severity measures. As a surrogate for severity for COPD, we controlled for Elixhauser comorbidity measures in our analysis. In addition, our study did not examine care coordination, such as early discharge to pulmonary rehabilitation, or nurse or self-management education [39,40]. Fourthly, some of the unadjusted comparisons of patient characteristics were statistically significant with a marginal clinical significance because of the large numbers. Lastly, our objective was to assess COPD-related ED visits and associated healthcare utilization. Some patients may have had visits to urgent care or other ambulatory care sites; this would have led to an underestimation of healthcare utilization for AECOPD. However, because we focused on the characteristics and burden of the frequent ED utilizers for COPD, our observations are of direct relevance to the development of strategies to improve COPD care in this important patient population.

## Conclusions

By using large all-payer databases from two geographically dispersed states in US, we found that 29% of the ED patients with AECOPD had frequent ED visits and that lower socioeconomic status were significantly associated with a higher frequency of ED visits. This population accounts for the majority of all COPD-related ED visits, hospitalizations, near-fatal events, and hospital charges, and present an important public health challenge. Our observations provide a strong foundation for clinicians and researchers to evaluate specific phenotypes of COPD (e.g., frequent ED users) and to develop targeted preventive interventions. For policy makers, our findings underscore the importance of integrated strategies aimed at reducing COPD-related healthcare utilizations in an already-stressed healthcare system.

## Abbreviations

AECOPD: Acute exacerbation of chronic obstructive pulmonary disease; AHRQ: Agency for Healthcare Research and Quality; COPD: Chronic obstructive pulmonary disease; ED: Emergency department; HCUP: Healthcare Cost and Utilization Project; ICD-9-CM: International Classification of Diseases, Ninth Revision, Clinical Modification; SEDD: State Emergency Department Databases; SID: State Inpatient Databases.

## Competing interests

Dr. Camargo has provided consultation for AstraZeneca, Genentech, GlaxoSmithKline, Merck, Novartis, and Pfizer, and received research grants from AstraZeneca and Novartis. The other authors have no financial relationships relevant to this article to disclose.

## Authors’ contributions

Conception and design: KH, YT, and CC; Analysis and interpretation: KH, YT, CT, DB, and CC; Drafting the manuscript for important intellectual content: KH, YT, CT, DB, and CC. All authors read and approved the final manuscript.
